# On the Morbid Histology and Bacteriology of Equine Pneumonia

**Published:** 1895-07

**Authors:** Cecil French

**Affiliations:** M’Gill University and Royal Veterinary High School, Munich


### ON THE MORBID HISTOLOGY AND BACTERIOLOGY OF EQUINE PNEUMONIA.

BY CECIL FRENCH, D.V.S.,
M'GILL UNIVERSITY AND ROYAL VETERINARY HIGH SCHOOL, MUNICH.

In order to render clear and comprehensive to oneself a description of the minute pathological changes concurrent with those of a macroscopic nature in a pneumonitic lung, a thorough appreciation of the normal histological appearance of lung-tissue is essential.

It will be remembered that the bronchioles split up and become narrower and more divergent, and are lined by columnar epithelium until a short distance before they enter the terminal alveoli or air-sacs (the word "sac" is to be preferred to "cell," since the latter is apt to be confused with that designating the vital elementary structures lining the cavity). Here the passage becomes again dilated to form an infundibulum which merges into the ultimate air-sac. In the smallest bronchioles the muscular elements are lost and the epithelium become more cubical, gradually assuming a flattened appearance toward the alveoli. The elastic fibres form the most important basal tissue of the terminal sacs, and contiguous to the alveoli lie the capillaries and lymphatics.

Inflammations of the lungs (pneumonia) may be either acute or chronic, with well-marked differences, the former usually affecting a whole lobe (lobar) when it is of a croupous type, the latter certain lobules (lobular) when it is generally catarrhal in nature and concomitant with evidences of bronchitis and bronchiolitis. To these may be added the interstitial or fibroid varieties, distinguished by great thickening of the interstitial connective tissue consequent on some chronic irritation such as of tubercles or of the various pneumonokionoses, these latter being rare in the horse, and hypostatic pneumonitis, which occurs as a result of cardiac failure or incompetency during the course of some exhausting disease. Such inflammations may start from (*a*) the bronchioles, (*b*) the pleura, extending inward along the lymphatics, (*c*) the small capillaries, in cases of pyæmia (infarcts of microbes).

Three distinct degrees of inflammatory action may be recognized: simple congestion, congestion and exudation of leucocytes, congestion and exudation of leucocytes and red cells.

In a simple congestion the capillaries surrounding the alveoli are seen to be abnormally distended and filled with red cells, and, as in all inflammations, the blood current stagnates, and, according to one theory, produces a starvation of the capillary walls and of the highly-functional air-cells lining the alveoli. These now lose control of their secretory power, and are unable to prevent a transudation into the sacs of certain of the blood fluids. Or, from another point of view, we may regard this outpouring of fluid as an attempt on the part of the organism to flush certain morbid areas.

The process may now go on to the second stage, that of diapedesis of leucocytes and denudation of the air-cells.

So far the differences between the catarrhal and primary stages of the croupous forms would seem only apparent as regards acuteness and intensity of the congestive processes. In the former the congestion is more of a sluggish circulation, the mucous membrane exhibiting a depraved state. The bronchial cells proliferate and are cast off, and on the extension of the congestion to the alveoli the flat air-cells also swell out and migrate. In this form can be seen numbers of the large "catarrhal" cells, regarded by some as being entirely the denuded swollen air-cells, by others as wandering leucocytes.

In the croupous type a further and more severe stage is reached, that of hemorrhagic exudation, or exodus of red cells. Coagulation then takes place, the plugs adhering to the walls of the alveoli. This is the stage of red hepatization, rarely seen, as death seldom occurs at this period. Ths hæmoglobin dissolves out of the red cells, developing into an insoluble pigment known as hæmosiderin, and leaves them of a pale appearance. Large numbers of leucocytes pass out, the fibrin contracts, the clot becomes looser, breaks up, and degenerates, assuming a granular character. This is the stage of gray hepatization, which passes insensibly into that of resolution, characterized by expectoration and absorption and regeneration of the lining air-cells. The large catarrhal cells are actively engaged in taking up the hæmosiderin, some then to degenerate themselves, others to return to the neighboring lymphatics. A similar notable phagocytic performance is witnessed in the activity of the leucocytes in the tonsils in man, which pass out to devour the inhaled bacteria.

Equine pneumonia is apparently dependent for a causative agent on a specific micro-organism not unlike that found in the human pneumonitic lung, but nevertheless different in that it possesses distinctive biological characters. The micrococcus pneumoniæ crouposæ of man takes the Gram stain, whereas that of the horse does not; the former will not grow at the ordinary room temperature, the latter will.

Schütz obtained and described the bacterium pleuro-pneumoniæ equi in 1887, though it had previously been observed by Friedberger in 1873, by Leyden, Mendelsohn, and Peterlein in 1884, and by Perroncito in 1885. In shape it is oval, undergoing division at right angles to the long axis, thereby lying in pairs and resembling somewhat the bipolar bacteria of fowl-cholera, though smaller, but claimed by some to be identical.

The secondary necrosis of the lung-tissue is due to other microphytes, which induce putrefaction as soon as pneumonic processes have disturbed the lung-tissue. To obtain a pure culture of the microbe direct from the lung is very difficult. Perfectly sterile instruments only must be used, and the tissue teased apart until one of the alveolar fibrinous plugs be isolated. From this a gelatin culture may be made, but as the various secondary necrotic bacteria usually have time to generate and multiply in great numbers before the dissection can be made, a very mixed growth is the result. But by subcutaneously inoculating a white mouse with the material, the pneumonia microbes will very often thrive rapidly to the exclusion of the others, and kill the subject in from twenty-four to forty-eight hours. Then from all parts of the body a pure culture can easily be obtained.

